# Accidental methanol ingestion: Case report

**DOI:** 10.1186/1471-227X-10-3

**Published:** 2010-02-22

**Authors:** Jelle L Epker, Jan Bakker

**Affiliations:** 1 Erasmus MC Rotterdam, Department of Intensive Care Medicine, PO Box 2040, 3000 CA Rotterdam, the Netherlands

## Abstract

**Background:**

The incidence of methanol (CH_3_OH) intoxication differs enormously from country to country. Methanol intoxication is extremely rare in the Dutch population. Even a low dose can already be potentially lethal. Patients are conventionally treated with hemodialysis. Therefore we'd like to present a report of a foreign sailor in Rotterdam who accidentally caused himself severe methanol intoxication, with a maximum measured concentration of 4.4 g/L.

**Case presentation:**

The patient presented with hemodynamic instability and severe metabolic acidosis with pH 6.69. The anion gap was 39 mmol/L and the osmol gap 73 mosmol/kg. Treatment with ethanol and continuous venovenous hemodiafiltration (CVVH-DF) was initiated. Despite the hemodynamic instability it is was possible to achieve rapid correction of pH and methanol concentration with CVVH-DF while maintaining a stable and therapeutic ethanol serum concentration. Despite hemodynamic and acid-base improvement, our patient developed massive cerebral edema leading to brain death. Permission for organ donation was unfortunately not ascertained.

**Conclusions:**

We conclude that in a hemodynamic instable situation high methanol concentrations and methanol-induced derangements of homeostasis are safely and effectively treated with CVVH-DF and that severe cerebral edema is another possible cause of death rather than the classical bleeding in the putamen area.

## Background

The nature and incidence of alcohol intoxications are race, sex, culture and geographical localization dependent in a lot of cases [1-3]. Since methanol is not readily available and since there's no culture of distilling alcohol at home, severe methanol intoxications are extremely rare in the Netherlands. A foreign sailor visiting the Netherlands accidentally caused himself severe methanol intoxication by drinking unregistered illegally bought industrial alcohol. The background of the patient combined with the particular chemical derangements was indicative of potential methanol intoxication [4,5].

Hemodialysis in combination with ethanol or fomipezole, a costly but powerful alcohol dehydrogenase (ADH) blocker is the first choice treatment in case of a severe intoxication [6,7]. Due to severe hemodynamical instability hemodialysis was not an option and fomipezole was not available. Therefore the patient was treated with CVVH-DF and ethanol infusions to block the ADH. In the literature just a few cases using CVVH-DF for the treatment of methanol intoxication have been published so far [8,9]. Despite the fact that CVVH-DF is a second choice treatment, the metabolic derangements and the hemodynamic parameters improved rapidly after fluid resuscitation and initiation of CVVH-DF. Unfortunately the patient developed signs of cerebral herniation after all parameters had normalized. The CT scan showed instead of bleeding in the putamen massive cerebral edema followed by brain death of the patient.

In case of hemodynamical instability in a patient with methanol induced metabolic derangements, CVVH-DF in combination with ethanol infusion is a relatively cheap, save and effective alternative for hemodialysis and fomipezole.

## Case Presentation

A 26-year-old foreign sailor was admitted to our emergency department because of hypothermia and low Glascow Coma Scale (GCS). The patient was found unconscious in his cabin by the ship's captain, after not appearing on deck for his shift. One of his colleagues confessed they had been celebrating together about 8-12 hours ago at the end of their shift, with alcohol they bought illegally in a small harbor store the day before.

On the emergency department we saw an unconscious Caucasian male, bodyweight 68 kg's with a maximum GCS of 3 and a body temperature of 35°Celsius. Pupils were reactive to light on both sides. Initial blood pressure was 80/40 mm/Hg with a regular heartbeat of 126 beats/min. Respiration rate was 30, but shallow. Auscultation of the lung was normal. The heart sounds showed no abnormalities, except for a mild systolic (grade II/VI) murmur. The remaining physical examination was normal.

Laboratory results showed a severe metabolic acidosis with a HCO_3_^- ^of 4.2 mmol/L and pH of 6.69. The serum osmolality was 379 mosmol/kg, Na^+ ^146 mmol/L, K^+ ^7.7 mmol/L, Urea 5.8 mmol/L, Glucose 4.6 mmol/L, Cl^- ^111 mmol/L and Lactate 11.2 mmol/L.

Immediately after arrival the patient was intubated and central venous access was obtained.

**Figure 1:**
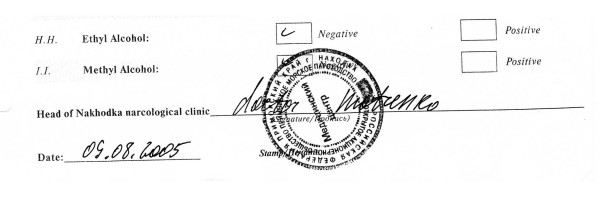
**Official proof of negative alcohol screening**.

Because of the suspicious circumstances, the severity of disease, the depth of acidosis and the osmol, anion an bicarbonate gap of respectively 73 mosmol/kg, 39 mmol/L and 6, methanol or ethylene glycol intoxication was suspected, even though the patient carried a document with a negative toxicology screen, as proof of a life without drugs, ethanol and even methanol, that was signed for his contractor only a few weeks ago (Figure 1).

### Anion gap

([Na]+ [K]) - ([CL]+ [HCO_3_]) = (146+7.7) - (111+4.2) = 39 mmol/L

### Osmol gap

Serum osmol - (1.86 (Na+K) + glucose + urea + 10) = 379-306 = 73 mOsm/kg [10,11].

### Delta gap or Bicarbonate gap

(AG-Normal AG) - (Normal bicarbonate- [HCO_3_]) = (38-12) - (24-4) = 6 indicating an almost pure anion gap acidosis [5].

The patient was transferred to the ICU where CVVH-DF was promptly initiated combined with a continuous infusion of 22 grams ethanol per hour over a central venous catheter, after an i.v. loading dose of 62 grams. The hypotension was successfully treated with volume suppletion and norepinefrine with a maximum dose in the first hours of 1.57 microgram/kg/min. Folate and thiamine were also administered.

**Figure 2:**
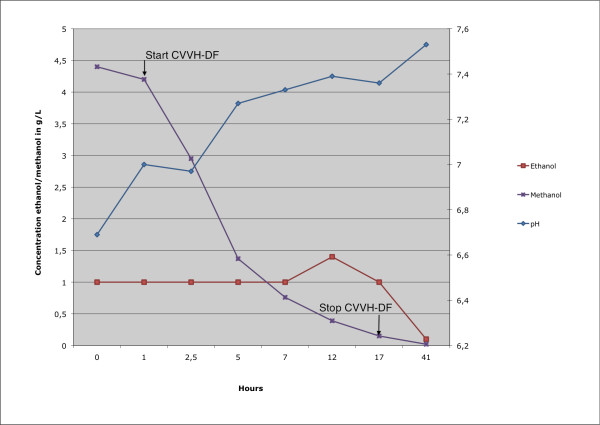
**pH, methanol and ethanol in g/L versus time**.

Following these measures the hemodynamic condition of the patient improved markedly. The pH and lactate levels normalized, as did the methanol concentration (Figure 2).

During the CVVH-DF we were able to maintain a stable serum ethanol concentration between 1-1.5 g/L.

**Figure 3:**
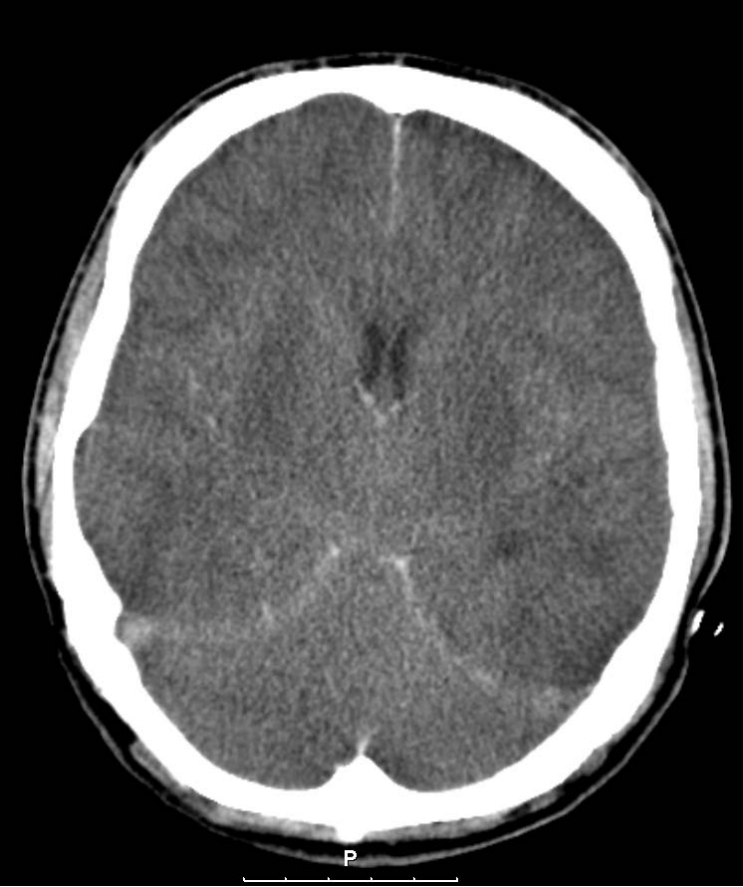
**Severe cerebral edema with compression of the ventricles**.

Although the hemodynamic parameters improved, the patient remained unresponsive and unconscious. Because of the initial high level of methanol and the severity of the acidosis, severe neurological damage was to be expected. Neurological examination showed signs of severe neurological damage like apnea, a negative vestibular caloric test and absence of the corneal and oculocephalic reflex. Our patient developed also diabetes insipidus at that time. A CT scan was made to visualize the nature and severity of the damage. This scan showed massive edema with diminishing grey- and white matter differentiation both supra and infra tentorial. The third and fourth ventricle as well as the basal cisterns were not identifiable anymore (Figure 3).

On the third day (48 hours after admission) 30 hours after the ethanol infusion and hemodialysis were discontinued, brain death was officially diagnosed.

Ethical approval was obtained from the Medical Ethics Committee of the Erasmus MC for publication of this report.

Methanol (CH_3_OH) intoxication has been a rare intoxication in the Dutch population over the years [12]. On the other hand, outbreaks of methanol intoxication, caused by illegally produced alcohol, have been reported extensively in some other countries. Due to progressive open market policy and the increase of free traffic of workers within and outside Europe, the incidence of alcohol intoxications could increase in countries that are not yet familiar with this problem.

Severe methanol intoxication is a rare but life-threatening event, even ingestion of a small amount of methanol can be potentially lethal [13,14]. Prompt action should therefore be taken when methanol intoxication is suspected, because delay can have deleterious consequences. Awareness of even the rare possibility of methanol ingestion is thus very important in emergency medicine. The symptoms of methanol intoxication are not very specific except for the visual disturbances and specially the so called "snowstorm vision" [15]. On the other hand, the presence of a high anion gap acidosis combined with a high osmol gap and normal Delta gap should raise the level of suspicion.

The normal delta- or bicarbonate gap in this case ruled out the presence of another, not directly detectable metabolic derangement, beside the already existing methanol-induced acidosis [5].

Though methanol itself is not very poisonous, the degradation products are extremely harmful. Methanol is easily and rapidly absorbed in the digestive tract and even through inhalation and skin absorption[16]. Methanol is transported to the liver where it is rapidly metabolized by ADH to formaldehyde, which is further converted into the toxic formic acid, by formaldehyde dehydrogenase (FDH). Eventually formic acid is converted into CO_2 _and H_2_O. Especially this last step is very important, because this is a slow, enzyme depended pathway, which causes accumulation of formic acid in already intoxicated humans. This last step is considered to be folate dependent, therefore administration of folate in formic acid intoxication has been advocated [17].

The first roughly estimated maximum methanol concentration in our patient, calculated with the use of serum osmolality in the presumed absence of ethanol was 2.5 g/L. The lower limit for methanol intoxication treatment is by tradition 0.2 g/L, although there's no clear empirical support for this value [7].

### Estimated Methanol concentration

Calculated osmol gap × 10^-3 ^× methanol molar mass = methanol concentration in g/kg = 73 × 10^-3 ^× 34.02 = 2.5 g/kg

Nevertheless the concentration was high enough to initiate CVVH-DF immediately. The, by gas chromatography measured methanol concentration, that was obtained later, was unfortunately much higher (4.4 g/L) and confirmed the absence of ethanol. The discrepancy between the calculated and measured methanol concentrations is retrospectively probably best explained by accidental dilution of the serum sample used. This sample, as became clear later, was drawn solely and separately for osmolality testing. Most likely it was drawn from an arterial line that was flushed or from an i.v. tube, which had been running with resuscitation fluids. Since no other tests were performed with this sample the possibility of a dilution error was not recognized.

Although hemodialysis is considered much more effective in clearing methanol, CVVH-DF was chosen because of the hemodynamic instability. The scarce literature available about the use of CVVH-DF in methanol intoxication suggests that it accelerates methanol elimination usefully, shortens the time to target serum methanol concentrations and likely shortens the period of metabolic derangement [9]. Classical hemodialysis, with an estimated clearance of 250 ml/min, is about 5 times more effective in clearing methanol than CVVH-DF with a clearance of maximal 50 ml/min [8].

The methanol elimination halve time (T_1/2_) using CVVH-DF as described by Kan et all is 10-12 hours following first order kinetics [9]. In our patient the methanol T_1/2 _in the presence of an adequate ethanol level was about 3.5 hours, and also followed first order kinetics. This remarkable short halve time can be explained by the aggressive fluid resuscitation that took place in the first hours, the well preserved kidney function of the patient and the much larger filter surface area: we used a 1.9 m^2 ^filter in contrast with he 0.6 m^2 ^filter described by Kan et all.

Because the same ADH competitively breaks down both ethanol and methanol, the administration of ethanol during methanol intoxication reduces the velocity of formic acid production. A concentration of 1 g/L ethanol is sufficient to fully block the degradation of methanol [18]. Since the elimination of methanol is otherwise slow, a steady ethanol concentration for a longer period of time is essential. The maintenance of a stable ethanol concentration especially in hemodynamic instable patients on dialysis is considered a challenge [19]! Despite the unstable situation we were able to gain and maintain a stable and therapeutical ethanol concentration until the methanol was fully washed out 16 hours after initiation of CVVH-DF.

Fomipezole a safe and highly effective ADH blocking drug, and an alternative for ethanol therapy, was not available [6].

Although the clinical parameters improved in our patient the neurologic signs deteriorated even after the alcohol concentrations were normalized. Initially the patient was comatose (GCS = 3) without signs of brainstem damage, but 44 hours later, he developed signs of cerebral herniation like: apnea, diminishing brainstem reflexes and diabetes insipidus.

The CT scan of the brain showed predominately signs of high intra cerebral pressure with occlusion of the third and fourth ventricle rather than bleeding and necrosis of the putamen and subcortical regions that has been described in severe cases [20]. Although organ donation after methanol intoxication was considered a serious option [21], family authorization, necessary because there was no donor declaration or written will available, could unfortunately not be obtained.

Ventilation- en vaso-active support was actively withdrawn thereafter.

The 81-year-old shopkeeper and reseller of the "alcohol" was, 8 months later charged and found guilty of involuntary manslaughter, because he had been unaware of the fact that he had been selling a potentially lethal alcohol like methanol. Taking into account his age, health status, the loss of his shop and the fact that he felt guilty, he was sentenced to a suspended term in jail and to community service in an old men's home for several weeks.

## Conclusions

◦ In the differential diagnosis of an emergency medicine accident, cultural background and behavior should always be taken into account.

◦ Methanol intoxication induced derangements of homeostasis are successfully treated with CVVH-DF and intravenous ethanol even in a hemodynamic instable patient.

◦ Methanol is safely and effectively cleared with CVVH-DF

◦ Metabolic improvements do not equal to healing the patient

◦ Signs of brain damage in methanol intoxication are not always based on necrosis and bleeding, but may also reflect severe brain edema.

◦ Do not sell what you don't' know yourself.

## List of abbreviations

GCS: Glascow Coma Score; CVVH-DF: Continuous VenoVenous Haemo-DiaFiltration; ADH: Alcohol Dehydrogenase; FDH: Formaldehyde Dehydrogenase.

## Consent

In this case the patient was unable to give consent and no family or proxy was available to give consent for publication.

Since in this case only retrospective data of an already deceased patient were used, who was treated according standards of normal care, no informed consent or ethical approval was necessary for publication according to Dutch law. To make sure that no ethical or legal rules were violated we additionally asked the Medical Ethics Committee of the Erasmus MC as an independent surrogate proxy for the patient. After a few adjustments in the text to secure privacy, the consent for publication was granted. A copy of this proxy consent is available for review by the Editor-in-Chief of this Journal.

## Competing interests

The authors declare that they have no competing interests.

## Authors' contributions

JLE treated the patient and wrote the case report, JB supervised the writing and made some major changes in manuscript after reviewing the first versions. Both authors read and approved the final manuscript.

## Pre-publication history

The pre-publication history for this paper can be accessed here:

http://www.biomedcentral.com/1471-227X/10/3/prepub
